# Pseudo-Harlequin syndrome: a case report

**DOI:** 10.1093/ehjcr/ytag298

**Published:** 2026-07-07

**Authors:** Álvaro Velasco, Roberto Jiménez Manso, Laura Domínguez Pérez, Roberto Martín Asenjo, Elena Puerto

**Affiliations:** Department of Cardiology, Hospital Universitario 12 de Octubre, Av. de Córdoba, s/n, Madrid 28041, Spain; Fundación para la Investigación Biomédica del Hospital Universitario 12 de Octubre (FIBH12O), Instituto de Investigación Sanitaria Hospital 12 de Octubre (imas12), Av. de Córdoba, s/n, 28041, Madrid, Spain; Department of Cardiology, Hospital Universitario 12 de Octubre, Av. de Córdoba, s/n, Madrid 28041, Spain; Department of Cardiology, Hospital Universitario 12 de Octubre, Av. de Córdoba, s/n, Madrid 28041, Spain; Department of Cardiology, Hospital Universitario 12 de Octubre, Av. de Córdoba, s/n, Madrid 28041, Spain; Department of Cardiology, Hospital Universitario 12 de Octubre, Av. de Córdoba, s/n, Madrid 28041, Spain

**Keywords:** Cardiogenic shock, ECMO, Harlequin syndrome, Case report

## Abstract

**Background:**

This case describes a teenager with Danon disease, severe biventricular dysfunction (INTERMACS 3), and combined, reversible pulmonary hypertension, who was receiving ambulatory levosimendan while awaiting elective heart transplantation when he developed refractory shock.

**Case summary:**

A 16-year-old male was transferred in SCAI-D cardiogenic shock despite high-dose vasoactive therapy, with rising lactate and acute kidney injury. A multidisciplinary Heart/Shock team indicated hybrid central–peripheral veno-arterial-venous extracorporeal membrane oxygenation (ECMO) with left ventricle unloading. After initial stabilization, he developed falling ECMO flows, increasingly negative drainage pressures, hypotension, oliguria, and lactate elevation. Echocardiography revealed collapsed ventricles and mild pericardial effusion, but tamponade seemed unlikely due to an open pericardium. Progressive upper-body congestion and cyanosis later appeared, mimicking Harlequin syndrome. Surgical exploration revealed circumferential pericardial clots causing tamponade. Their removal resulted in immediate perfusion, ECMO flow, and organ recovery.

**Discussion:**

This represents an atypical tamponade presentation under ECMO, mimicking Harlequin physiology. We propose the term ‘pseudo-Harlequin syndrome’. Suspicion for tamponade must remain high in ECMO patients, even when classical signs are absent.

Learning points

- Cardiac tamponade in extracorporeal membrane oxygenation (ECMO) may lack classical clinical or echocardiographic signs, so it is still a clinical diagnosis.
- Unusual patterns of differential congestion or discolouration in ECMO patients should prompt re-evaluation of mechanical and anatomical complications.

## Summary figure

**Figure:**
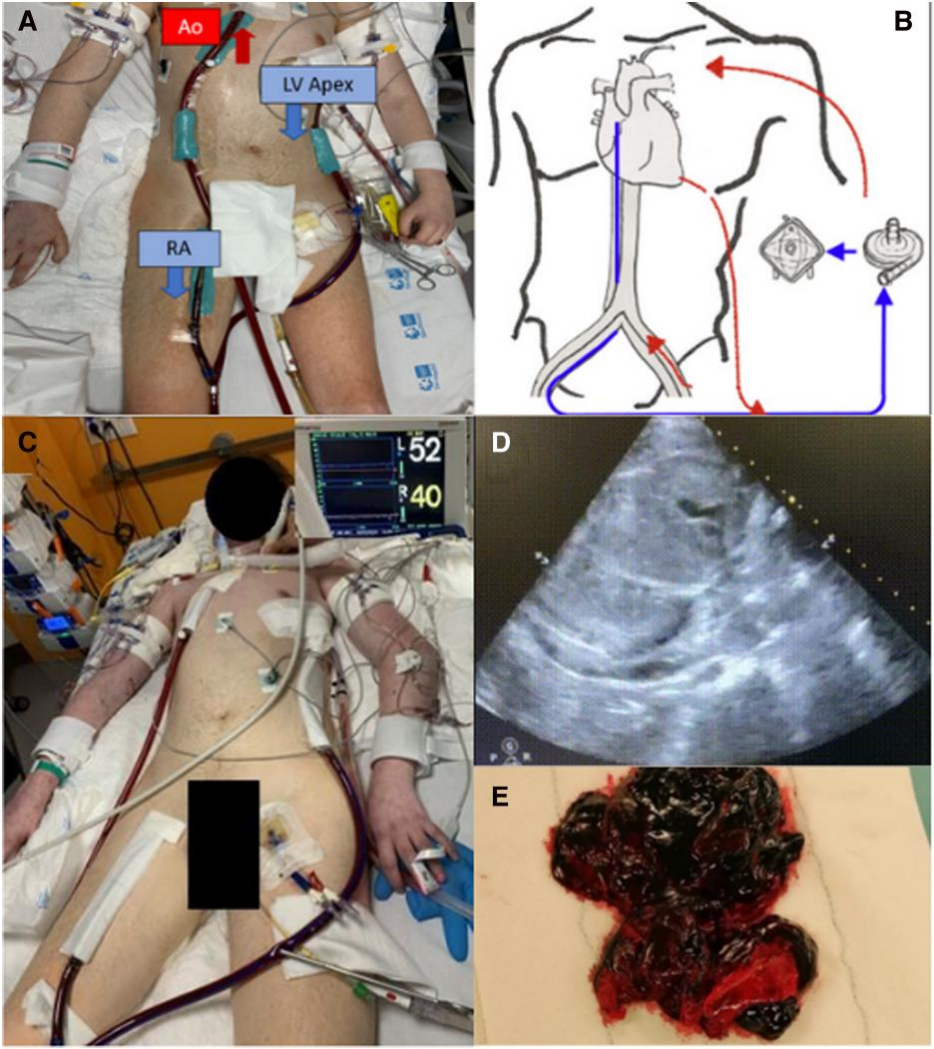
(A) Patient prior to the intervention, with cyanotic colouration in the upper limbs and pale colouration from the mammary region to the lower limbs. Note the cerebral hypoperfusion detected with the non-invasive cerebral oximeter INVOS. (B) Extracorporeal membrane oxygenation (ECMO) circuit diagram. Drainage cannulas from right atrium (RA, blue arrow) and from left ventricle (LV) apex (blue arrow), joined in a Y-shaped circuit. Return cannula in the aorta (Ao). (C) Patient after surgery, with recovery of skin colouration and improvement in cerebral oxygenation value measured by INVOS. (D) Transthoracic echocardiogram. Short axis showing the completely collapsed cavities of the left ventricle (LV) and right ventricle (RV). (E) Pericardial clots surgically removed via thoracotomy.

## Case report

We present the case of a 16-year-old male with Danon disease (pathogenic mutation in the *LAMP2* gene), presenting with non-obstructive hypertrophic cardiomyopathy with severe biventricular dysfunction, classified as advanced heart failure (INTERMACS 3),^1^ receiving ambulatory cycles of levosimendan while awaiting elective heart transplantation.

The patient also had combined, reversible Group 2 pulmonary hypertension (mean PAP 59 mmHg, PCWP 33 mmHg). He previously had a CRT-D (Cardiac Resynchronization Therapy with Defibrillator) due to complete atrioventricular block.

## Initial work-up

The patient was transferred to our centre from a secondary hospital due to refractory decompensated heart failure, where he received intravenous diuretics and vasoactive support for 5 days.

Upon arrival at the Cardiovascular Critical Care Unit, he was receiving noradrenaline 0.2 mcg/kg/min and dobutamine 8 mcg/kg/min. The physical examination revealed hypotension (90/40 mmHg), delayed capillary refill, and pale skin and mucous membranes. No murmurs were detected. Bilateral crackles were noted on lung auscultation, with minimal oedema in the lower limbs.

An urgent blood gas analysis was performed, highlighting a venous lactate level of 4.0 mmol/L. Laboratory tests revealed acute kidney injury, with creatinine rising to 2.5 mg/dL from baseline. According to current guidelines, a diagnosis of SCAI-D cardiogenic shock was established due to lack of response to increased vasoactive drug support.^1^

## Diagnosis and management

Due to the refractory nature of the shock, early implantation of biventricular short-term mechanical circulatory support, as recommended in ESC guidelines, was decided in the Heart/Shock team meeting, which included cardiologists, cardiac surgeons, and anaesthesiologists.

Veno-arterial-venous ECMO was configured using a hybrid configuration of central and peripheral cannulation. This included a percutaneous drainage cannula in the right femoral vein, a direct apical cannula in the LV for unloading, and a central return cannula in the ascending aorta (*Summary Figure, A and B*). The procedure—thoracotomy with open pericardiotomy into the pleural space—was uneventful.

After ECMO implantation, the patient was supported with an ECMO blood flow of 3.1 L/min (3500 rpm), dobutamine (4.5 mcg/kg/min), nitroprusside (0.9 mcg/kg/min), and invasive mechanical ventilation. He remained stable, with a mean arterial pressure (MAP) of 80 mmHg, adequate tissue and organ perfusion (normal lactate levels), and good urine output (300 mL/h). The ECMO drainage pressure was −13 mmHg, and central venous pressure (CVP) was 7 mmHg.

**Figure 1:**
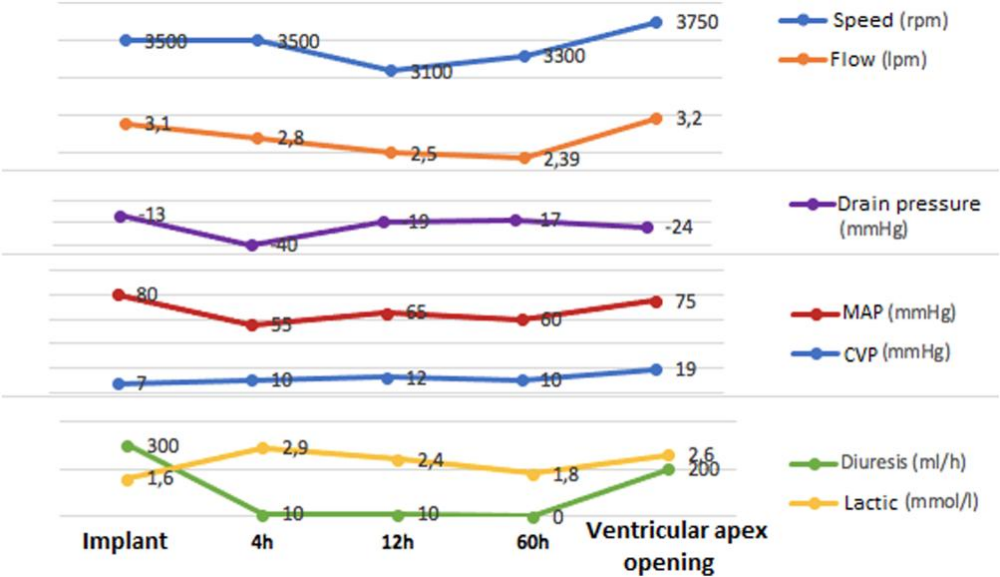
Temporal evolution of ECMO speed and flow values, along with drainage pressures, mean arterial pressure, central venous pressure, diuresis, and lactic acid.

In the following hours, despite maintaining the same ECMO speed, there was a drop in blood flow and a decrease in MAP to 55 mmHg, followed by a rise in CVP. This led to near-anuria and an increase in lactate to 2.9 mmol/L. These changes were accompanied by a sharp drop in ECMO venous pressure, becoming more negative, reaching −40 mmHg (see *Figure 1*).

## Follow-up

Due to the haemodynamic instability, a bedside transthoracic echocardiogram was performed, revealing a completely collapsed LV and right ventricle (*Summary Figure D*), along with a mild, echo-dense global pericardial effusion and a dilated inferior vena cava (21 mm) without inspiratory collapse. Transoesophageal echocardiography confirmed these findings. Both venous 23F cannula malposition [potentially obstructing superior cava vein (SVC) inflow] and aortic 15F cannula malposition (leading to a pseudo-Harlequin phenomenon with preferential lower-body perfusion) were carefully considered as common causes of upper-body oedema and cyanosis. However, transoesophageal echocardiography confirmed adequate positioning of femoral venous cannula at SVC-RA junction with no evidence of obstruction to SVC inflow or preferential streaming, and no malposition of the aortic cannula was suspected.

Tamponade was suspected and discussed with the multidisciplinary Heart/Shock team. However, since right atrial pressure was not markedly elevated, there were no significant suction signs in the inflow cannula, and the pericardium was open, tamponade was initially ruled out. Excessive biventricular unloading was considered the most likely cause.

In response, volume resuscitation was initiated, and ECMO speed was reduced to 3100 rpm, resulting in reduced flow and a less negative drainage pressure (−19 mmHg). Nitroprusside was discontinued, and dobutamine was titrated up to 9 mcg/kg/min due to hypotension. This led to a slight improvement in MAP, but the patient remained oliguric (*Figure 1*).

The patient remained relatively stable with minor adjustments to vasoactive medications over the next 60 h. However, progressive congestion of the head and upper extremities developed, accompanied by upper-body cyanosis, while the lower body (from the nipple line downward) remained pale *Summary Figure A*). This cyanosis was confirmed by regional differences in saturation O_2_ and INVOS values, as shown in *Summary Figure A*. These findings were consistent with Harlequin syndrome, which is highly unusual in central ECMO with preserved lung function.

At this point, the possibility of cardiac tamponade was reconsidered, with drainage pressures reaching −17 mmHg.

Surgical intervention was eventually undertaken. Intra-operatively, circumferential clots causing cardiac tamponade were discovered (*Summary Figure E*) and removed without complications.

Post-operatively, the patient exhibited immediate improvement in skin colouration and resolution of upper-body congestion. Extracorporeal membrane oxygenation speed was increased to 3700 rpm, with a corresponding rise in flow to 3.2 L/min, resulting in significant recovery in urine output. Cerebral perfusion also improved, as evidenced by cerebral oximetry comparisons (*Summary Figure A and C*). The patient’s haemodynamic status improved steadily, allowing gradual reduction of vasoactive support and eventual extubation.

## Discussion

Cardiac tamponade in ECMO patients is a rare complication (2.6%) according to the largest registry to date, which includes 84 430 patients.^2,3^ However, it is a potentially life-threatening condition that requires prompt diagnosis. Cardiac tamponade in ECMO does not present with the classical clinical or haemodynamic signs observed in non-ECMO patients, as physiology is significantly altered. Furthermore, echocardiographic assessment is made more challenging by the presence of central cannulas and pericardial space instrumentation.^4^

The dramatic skin discolouration initially suggested Harlequin syndrome (or North-South syndrome), typically seen in peripheral Veno-arterial ECMO with poor pulmonary function. In such cases, desaturated blood from the failing lungs reaches the coronary and cerebral circulations.^5^ However, this syndrome has not been described in central ECMO, which is one of its treatment options.

In our case, the clinical picture mimicked this syndrome. Therefore, we propose the term pseudo-Harlequin syndrome to describe such a presentation. These skin changes and upper-body congestion are not typically seen in standard cardiac tamponade without mechanical circulatory support, likely due to earlier diagnosis and intervention. We hypothesize that pericardial clots compressing the RA impeded proper venous return. The SVC is particularly vulnerable due to its limited valvular support, unlike the inferior vena cava, which benefits from a valved venous system. Overall, the most suitable pathophysiological explanation we find is that these clots produced a SVC syndrome. As far as we are concerned, this is the first case reported of SVC syndrome in adult on central ECMO.

In cases of tamponade under ECMO support, a gradual trend towards more negative drainage pressures along with rising CVP is a commonly described haemodynamic pattern. However, our patient deviated from this pattern, and to our knowledge, no similar presentations have been reported to date.

Thus, we emphasize that in ECMO-supported patients, clinical suspicion of pericardial tamponade must take precedence, even in the absence of classical haemodynamic signs.

## Supplementary Material

ytag298_Supplementary_Data

## Data Availability

The data underlying this article are fully available in the article.

## Lead author biography

I am a cardiology resident at 12 de Octubre University Hospital. I am interested in cardiology critical care area, preventive cardiology and interventional cardiology.

## Notes

**Consent:** Written informed consent was obtained from the patient for publication of this case report and any accompanying images.
